# Lens dislocation, OCTA, and archived *FBN1* reports: An exploratory study

**DOI:** 10.1371/journal.pone.0361193

**Published:** 2026-10-23

**Authors:** Muhammer Özgür Çevik, Metehan Simsek, Sadik Gorkem Cevik, Irfan Perente

**Affiliations:** 1 Department of Medical Genetics, Adiyaman University Faculty of Medicine, Adiyaman, Türkiye; 2 Department of Ophthalmology, Beyoglu Eye Training and Research Hospital, Istanbul, Türkiye; 3 Department of Ophthalmology, Bursa Yüksek İhtisas Training and Research Hospital, Bursa, Türkiye; Aravind Eye Care System, INDIA

## Abstract

**Purpose:**

Lens dislocation (ectopia lentis) is a recognized anterior-segment manifestation of fibrillinopathy. This exploratory phenotype-first study compared macular optical coherence tomography angiography (OCTA) parameters between participants with and without documented lens dislocation and descriptively reviewed available archived clinical *FBN1* reports.

**Methods:**

This retrospective, availability-based study included 16 participants classified by verified current or historical lens-dislocation status. Participant-level superficial capillary plexus (SCP) vessel density was calculated by averaging valid bilateral measurements or using the single valid eye. Five OCTA endpoints were compared using two-sided Mann–Whitney U tests; Benjamini–Hochberg q values were used as a multiplicity sensitivity analysis. Seven archived clinical *FBN1* reports from lens-dislocation-positive (LD+) participants were summarized without variant reclassification. No genotype–OCTA association was assessed.

**Results:**

The cohort comprised 7 LD+ and 9 lens-dislocation-negative (LD−) participants; 15 participants (6 LD+ and 9 LD−) contributed quantitative OCTA data. Foveal SCP vessel density was lower in LD+ participants (median [IQR], 19.38% [18.68–20.04] vs 30.20% [29.20–31.85]; p = 0.036), but the finding was not retained after multiplicity sensitivity analysis (q = 0.180). Whole-image, parafoveal, and perifoveal vessel-density comparisons were not significant (p = 0.328, 0.272, and 0.238; q = 0.410 each), and OD-only foveal avascular zone area did not differ (n = 14; p = 0.606; q = 0.606). Six of seven archived reports documented an *FBN1* variant; one was negative within its assay scope.

**Conclusions:**

The unadjusted foveal signal was not robust to multiplicity analysis, and other OCTA endpoints did not differ significantly. Marked between-group differences in spherical equivalent and axial length, the small sample, and heterogeneous routine-care genetic reports limit interpretation. These findings are hypothesis-generating and do not establish OCTA as a diagnostic or prognostic test or demonstrate an individual-level genotype–OCTA association.

## Introduction

Lens dislocation (ectopia lentis) is a readily recognizable and clinically actionable anterior segment sign which may provide the presenting clue to an underlying systemic connective tissue disorder. The ophthalmologist might be the first clinician to suspect the disorder and initiate systemic and genetic evaluation, as it can be detected prior to molecular confirmation [1]. Such a sequence is especially important in pediatric and young adult eye care, where a striking anterior segment finding may lead to targeted genetic testing before a clear diagnosis of a syndrome is made [2,3].

*FBN1* encodes fibrillin-1, a major microfibrillar component of the ciliary zonule and extracellular matrix [4–7]. Pathogenic *FBN1* variation can cause Marfan syndrome and related fibrillinopathies, including presentations dominated by ectopia lentis [8–10]. Fibrillin-rich microfibrils also contribute to extracellular-matrix organization and latent TGF-β complex sequestration, providing biological context for systemic vascular manifestations [5,6,11,12].

Optical coherence tomography angiography (OCTA) is a non-invasive, dye-free modality that provides depth-resolved visualization of the retinal microvasculature and quantitative measures including vessel density and foveal avascular zone area [13]. Layer-specific OCTA measurements can be used to investigate subclinical microvascular variation, although they are influenced by device, image quality, segmentation, refractive status, and ocular biometry [14–19]. Prior studies in genetically confirmed Marfan syndrome reported differences in retinal vessel-density measures, but their reproducibility and diagnostic relevance in phenotype-first cohorts remain uncertain [3,20,21].

Recognizing ectopia lentis as an actionable clue to systemic connective-tissue disease, this exploratory, availability-based retrospective study compared macular OCTA parameters between groups defined by lens-dislocation status. Archived clinical genetic reports, when available, were reviewed descriptively for *FBN1* findings. The OCTA and genetic components were interpreted in parallel, yielding complementary, hypothesis-generating observations to inform future prospective studies of combined ophthalmic and molecular assessment.

## Methods

### Study design, setting, and ethics

This exploratory, availability-based retrospective cross-sectional study used clinical records and macular optical coherence tomography angiography (OCTA) images acquired during routine care at the Department of Ophthalmology Retina Unit of Bursa Yüksek İhtisas Training and Research Hospital, Bursa, Türkiye, between 1 January 2016 and 31 December 2020. The primary analysis compared participants according to documented lifetime lens-dislocation status, irrespective of molecular confirmation of Marfan syndrome or another fibrillinopathy.

The study was approved by the Bursa Yüksek İhtisas Training and Research Hospital Clinical Research Ethics Committee (approval no. 2011-KAEK-25 2021/08–25; 11 August 2021) and was conducted in accordance with the Declaration of Helsinki. The ethics committee waived the requirement for informed consent because the study used existing clinical records and imaging data. Records and images were accessed for research purposes between 01/09/2021 and 01/12/2021 and were coded and de-identified before analysis.

### Participants and data structure

Potentially eligible participants were identified from the retina unit’s existing clinical files in which lens-dislocation status had been evaluated or documented. These records were cross-referenced with archived 6 × 6-mm macular OCTA examinations obtained during routine care. Eligibility required verifiable current or historical lens-dislocation status and at least one available macular OCTA record. Availability or content of genetic reports did not influence participant identification, eligibility, or lens-dislocation classification.

Eligibility was not restricted to participants with a molecularly confirmed diagnosis, and no additional diagnosis-based exclusion criterion was applied during retrospective selection. Because a complete screening denominator and contemporaneous screening or exclusion log were unavailable, sampling was availability-based rather than consecutive; denominator-level exclusion counts were therefore not inferred. No a priori sample-size calculation was performed because the study used the complete availability-based cohort meeting the retrospective eligibility criteria.

Sixteen participants met the eligibility criteria and formed the imaging cohort. Image quality was evaluated separately for each eye, whereas quantitative comparisons were conducted at the participant level to avoid treating two eyes from the same participant as independent observations.

### Lens-dislocation phenotype and clinical variables

Participants were classified as lens-dislocation positive (LD+) if current or previously documented crystalline lens dislocation was present and as lens-dislocation negative (LD−) if no current or historical lens dislocation was documented. Classification was adjudicated through review of available ophthalmology and clinical records, including lifetime lens-dislocation history, laterality, current lens status, and previous lens surgery. Previous lens surgery and current lens status were recorded separately and did not replace the lifetime phenotype definition. Seven participants were classified as LD+ and nine as LD − .

Refraction and axial length were recorded separately for the right (OD) and left (OS) eyes. Objective refraction was measured using a Canon RK-F2 autorefractor/keratometer (Canon Inc., Tokyo, Japan), and spherical equivalent was calculated as sphere plus one-half cylinder. Axial length was measured using a NIDEK AL-Scan optical biometer (NIDEK Co., Ltd., Gamagori, Japan). For participant-level summaries, valid bilateral values were averaged; a unilateral value was used when the fellow-eye value was unavailable. Source-verified spherical-equivalent and axial-length measurements were available for all 16 participants.

### OCTA acquisition and quality control

Macular OCTA imaging was performed using a spectral-domain RTVue XR Avanti system with AngioVue/AngioAnalytics software (Optovue Inc., Fremont, CA, USA; software version/build 2017.1.0.151). A 6 × 6-mm macular scan centered on the fovea was acquired for each eye. Flow was detected using split-spectrum amplitude-decorrelation angiography. Quantitative analyses were restricted to the manufacturer-defined superficial capillary plexus; deep capillary plexus and choriocapillaris measurements were not analyzed.

All scans underwent eye-level quality assessment before quantitative extraction. Foveal centration, automated segmentation boundaries, vascular continuity, motion artifact, and overall signal quality were assessed by a single experienced retina specialist (I.P.) who was masked to lens-dislocation classification. No manual segmentation correction was performed.

Scans were eligible for the primary analysis if they had an SSI of at least 50, adequate foveal centration, acceptable automated segmentation, preserved vascular continuity, and no substantial motion or other image artifact. Scans failing any of these criteria were excluded. Under this quality rule, 29 eyes from 15 participants contributed quantitative vessel-density measurements; one participant remained in the imaging cohort but had no eye meeting the quantitative-analysis criteria.

Vessel density was calculated automatically by AngioAnalytics as the percentage of the analyzed area occupied by flow-positive vascular pixels. Whole-image, foveal, parafoveal, and perifoveal superficial capillary plexus vessel-density values were transcribed from device-generated reports. FAZ area was measured in square millimetres using the AngioVue semiautomatic non-flow measurement tool.

For participant-level vessel-density analyses, valid OD and OS measurements were averaged when both eyes were analyzable; when only one eye was analyzable, the valid unilateral measurement was used. FAZ analysis was restricted to available OD measurements because the retained analytical export contained no OS FAZ values. Missing OS FAZ values were not inferred or imputed.

### Genetic report review

Genetic testing was not performed as part of the research protocol. Archived clinical *FBN1* reports issued during routine care by the hospital’s accredited medical genetics laboratory were reviewed retrospectively. Testing strategies reflected the original clinical indication and included broader *FBN1* next-generation sequencing in some participants and family-specific targeted testing in others.

Reported regions, coverage, and assay limitations were retained on a report-specific basis. A report-negative result was therefore interpreted only within the assay scope stated by the laboratory and was not considered equivalent to a uniform comprehensive negative result.

Variant nomenclature, zygosity, and laboratory interpretation were transcribed as reported. Variants were not independently reconstructed from raw sequence data or reclassified under ACMG/AMP criteria or subsequent *FBN1*-specific ClinGen specifications. Broader reports cited the *FBN1* reference transcript NM_000138 without a version suffix, whereas targeted familial reports did not specify a transcript. The source-report descriptions c.4349G > C (p.Cys1450Ser) and c.5368C > T (p.Arg1790*) were consequently retained without author-generated transcript conversion or reinterpretation.

Seven archived genetic reports were retained, all corresponding to unique participants in the LD+ subgroup. The reports were linked to coded participants through a confidential identity crosswalk and constituted an ancillary descriptive series nested within the imaging cohort. Because no corresponding report series was available for the LD− group, genetic findings were not compared by lens-dislocation status. Individual-level genotype–OCTA association analysis was not undertaken because the report series was small, included related participants, and comprised non-uniform testing strategies.

### Statistical analysis

Statistical analyses were performed using Python 3.12.13 with NumPy 2.3.5, pandas 2.2.3, and SciPy 1.17.0. Because of the small sample and the exploratory design, continuous variables were summarized as median and interquartile range and compared using two-sided Mann–Whitney U tests without relying on preliminary normality testing. Categorical variables were summarized as n (%) and analyzed using two-sided Fisher’s exact tests for 2 × 2 comparisons.

The participant was the unit of analysis. Valid bilateral OCTA measurements were averaged, and a valid unilateral measurement was used when only one eye was analyzable. FAZ analyses were restricted to available OD measurements. Missing values were not imputed. No observations were excluded as statistical outliers; exclusions were based only on the prespecified OCTA image-quality criteria.

Effect sizes for continuous comparisons were expressed as rank-biserial correlations with percentile-bootstrap 95% confidence intervals based on 100,000 resamples performed within the lens-dislocation groups (seed 20260807). Negative values indicate lower measurements in LD+ participants.

The five OCTA endpoints—whole-image, foveal, parafoveal and perifoveal vessel density, and FAZ area—were treated as exploratory. Unadjusted p values were reported as the primary results, and Benjamini–Hochberg q values across the five endpoints were calculated as a multiplicity sensitivity analysis. A two-sided p < 0.05 was considered the nominal threshold for the unadjusted exploratory analyses; Benjamini–Hochberg q values were interpreted as a multiplicity sensitivity analysis. No multivariable model was fitted because the sample size was insufficient for stable adjusted estimation. Analyses were conducted using the locked, de-identified dataset and finalized on 7 August 2026.

### Use of artificial intelligence tools

OpenAI ChatGPT and Microsoft Powerpoint were used to assist with the conceptual organization and layout planning of Fig 1; the scientific content and final figure were reviewed and approved by the authors. All interpretations, conclusions, limitations, and scientific claims represent the authors’ own assessment and responsibility.

**Fig 1:**
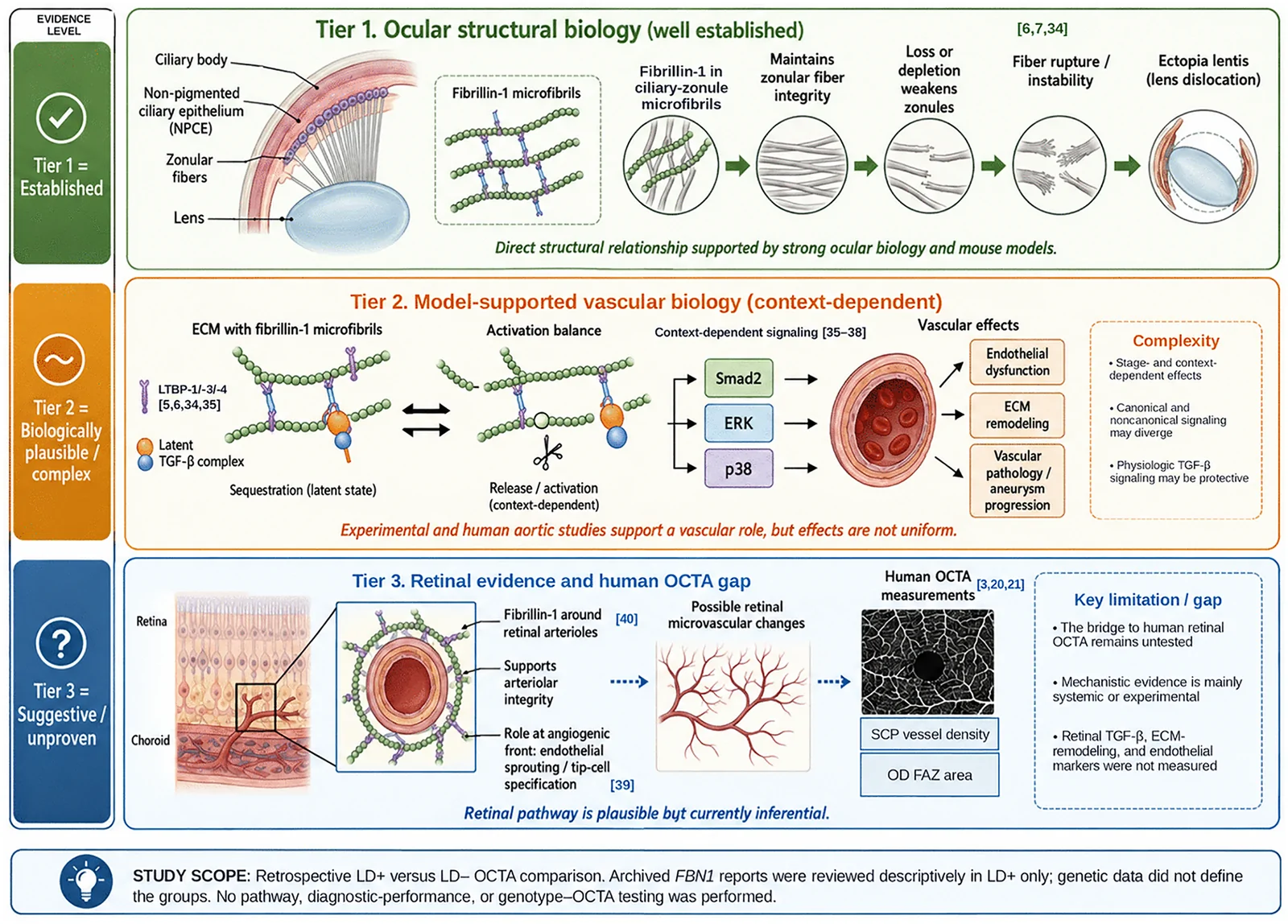
Three-tier evidence framework for ectopia lentis and retinal OCTA observations. Tier 1 summarizes the established structural role of fibrillin-1 in ciliary-zonule integrity [6,7,34]. Tier 2 summarizes context-dependent systemic and aortic evidence involving fibrillin-rich extracellular matrix and TGF-β regulation [5,6,34–38]. Tier 3 presents experimental retinal evidence and the unverified bridge to human optical coherence tomography angiography measurements [3,20,21,39,40]. The present study retrospectively compared OCTA parameters by lens-dislocation status and descriptively reviewed archived *FBN1* reports in the LD+ subgroup; it did not test the depicted pathways or individual-level genotype–OCTA associations.

## Results

The imaging cohort included 16 participants: 7 LD+ and 9 LD − . Median age was 11.0 years (IQR, 8.0–14.0) in LD+ and 14.0 years (IQR, 14.0–16.0) in LD− (p = 0.111). Females comprised 2/7 (28.6%) of LD+ and 5/9 (55.6%) of LD− participants (p = 0.358). Median SE was −7.75 D (IQR, −8.25 to −6.75) in LD+ and −1.50 D (IQR, −1.75 to −1.38) in LD− (p = 0.001); median AL was 25.25 mm (IQR, 24.80–25.60) and 23.65 mm (IQR, 23.55–23.65), respectively (p < 0.001). All participants were bilaterally phakic, and none had undergone previous lens surgery. Six of seven LD+ participants and all nine LD− participants contributed analyzable OCTA data. One LD+ participant contributed OS only because the OD scan had an SSI of 32, and one LD+ participant contributed no quantitative OCTA data because both eyes were below the prespecified SSI threshold. Baseline characteristics are shown in Table 1.

**Table 1:** Baseline clinical and imaging characteristics by lens-dislocation status.

Characteristic	LD+ (n = 7)	LD− (n = 9)	Unadjusted p value
**Age at OCTA, years**	11.0 (8.0 to 14.0)	14.0 (14.0 to 16.0)	0.111
**Female sex, n (%)**	2 (28.6)	5 (55.6)	0.358
**Spherical equivalent, D**	−7.75 (−8.25 to −6.75)	−1.50 (−1.75 to −1.38)	0.001
**Axial length, mm**	25.25 (24.80 to 25.60)	23.65 (23.55 to 23.65)	<0.001
**Bilaterally phakic, n (%)**	7 (100.0)	9 (100.0)	—
**Previous lens surgery, n (%)**	0 (0.0)	0 (0.0)	—
**Participants contributing quantitative SCP vessel-density data, n (%)**	6 (85.7)	9 (100.0)	0.438
**Participants with ≥1 eye excluded for image quality, n (%)**	2 (28.6)	0 (0.0)	0.175

Note: Values are median (interquartile range) or n (%). Spherical equivalent and axial length are participant-level values obtained by averaging valid bilateral measurements; a valid unilateral measurement was used when the fellow-eye value was unavailable. Continuous and categorical variables were compared using two-sided Mann–Whitney U and Fisher’s exact tests, respectively. P values are unadjusted and exploratory. —, not tested because there was no between-group variation. D, diopters; LD, lens dislocation; OCTA, optical coherence tomography angiography; SCP, superficial capillary plexus.

Participant-level SCP vessel-density medians were lower in LD+ than in LD− for all four regions (Table 2). Whole-image values were 45.48% (IQR, 44.31–49.45) versus 49.25% (IQR, 47.85–52.50; p = 0.328; q = 0.410). Foveal values were 19.38% (IQR, 18.68–20.04) versus 30.20% (IQR, 29.20–31.85; p = 0.036; q = 0.180). Although the foveal comparison met the conventional 0.05 threshold in the unadjusted exploratory analysis, it was not retained in the multiplicity sensitivity analysis. Parafoveal values were 48.35% (IQR, 47.02–53.88) versus 51.65% (IQR, 50.70–55.10; p = 0.272; q = 0.410), and perifoveal values were 45.20% (IQR, 43.56–50.44) versus 51.50% (IQR, 48.35–55.10; p = 0.238; q = 0.410). OD-only FAZ area was 0.148 mm^2^ (IQR, 0.145–0.432; n = 5) in LD+ and 0.272 mm^2^ (IQR, 0.252–0.312; n = 9) in LD− (p = 0.606; q = 0.606).

**Table 2:** Participant-level OCTA parameters by lens-dislocation status.

Parameter	LD+ median (IQR)	LD− median (IQR)	Rank-biserial correlation (95% CI)	Unadjusted p value	BH q value
Whole-image SCP vessel density, %	45.48 (44.31–49.45)	49.25 (47.85–52.50)	−0.33 (−0.89 to 0.33)	0.328	0.410
Foveal SCP vessel density, %	19.38 (18.68–20.04)	30.20 (29.20–31.85)	−0.67 (−1.00 to 0.00)	0.036	0.180
Parafoveal SCP vessel density, %	48.35 (47.02–53.88)	51.65 (50.70–55.10)	−0.37 (−1.00 to 0.33)	0.272	0.410
Perifoveal SCP vessel density, %	45.20 (43.56–50.44)	51.50 (48.35–55.10)	−0.39 (−0.93 to 0.26)	0.238	0.410
FAZ area, mm² (OD only)	0.148 (0.145–0.432)	0.272 (0.252–0.312)	−0.20 (−1.00 to 0.60)	0.606	0.606

Note: Values are median (interquartile range). For vessel-density endpoints, valid OD and OS values were averaged when both were available; a valid unilateral value was used otherwise (LD + , n = 6; LD − , n = 9). FAZ analysis was restricted to the available OD field (LD + , n = 5; LD − , n = 9). Missing values were not imputed. Unadjusted p values were obtained using exploratory two-sided Mann–Whitney U tests. Benjamini–Hochberg q values were calculated across the five OCTA endpoints as a multiplicity sensitivity analysis. Rank-biserial correlations are presented with percentile-bootstrap 95% confidence intervals; negative values indicate lower measurements in LD+ than in LD − . BH, Benjamini–Hochberg; CI, confidence interval; FAZ, foveal avascular zone; IQR, interquartile range; LD, lens dislocation; OCTA, optical coherence tomography angiography; OD, right eye; OS, left eye; SCP, superficial capillary plexus.

### Genetic findings

Seven archived clinical *FBN1* reports were mapped to seven unique LD+ participants in the imaging cohort. Six of seven reports (85.7%) documented an *FBN1* variant, whereas one report documented no disease-associated variant within its stated assay scope. Two distinct report-transcribed *FBN1* variants were documented: c.4349G > C (p.Cys1450Ser) in four related participants and c.5368C > T (p.Arg1790*) in two related participants. Four reports described broader *FBN1* NGS and three described family-specific targeted testing. These counts summarize unique participants and archived report findings; they do not represent independent variant discoveries or estimates of variant prevalence, diagnostic yield, or independent families. No genotype–phenotype or genotype–OCTA association was assessed (Table 3).

**Table 3:** Descriptive summary of archived clinical *FBN1* reports mapped to LD+ participants (n = 7).

Category	n (%)
**Reported findings**	
Reports documenting an FBN1 variant	6 (85.7)
c.4349G > C (p.Cys1450Ser)	4 (57.1)
c.5368C > T (p.Arg1790*)	2 (28.6)
No disease-associated FBN1 variant reported within the stated assay scope	1 (14.3)
**Testing approach**	
Broader FBN1 NGS reports	4 (57.1)
Family-specific targeted reports	3 (42.9)
**Total retained reports**	**7 (100.0)**

Counts represent unique participants, each with one retained archived report. Variant-specific rows are subsets of the six reports documenting an FBN1 variant and should not be added to the parent row. All reports were available for LD+ participants. Variant nomenclature and laboratory interpretations were transcribed as reported and were not independently re-derived or reclassified. Because participants included related individuals and testing strategies were heterogeneous, percentages are descriptive and should not be interpreted as variant prevalence or diagnostic yield. LD, lens dislocation; NGS, next-generation sequencing.

## Discussion

This exploratory, phenotype-first study combined an OCTA comparison stratified by documented lens-dislocation status with a descriptive review of archived clinical *FBN1* reports available for the LD+ subgroup. Of the 16 participants, 15 contributed quantitative vessel-density data and 14 contributed OD-only foveal avascular zone (FAZ) measurements. Median foveal superficial capillary plexus (SCP) vessel density was lower in the LD+ group in the unadjusted comparison (p = 0.036), but this finding was not retained after Benjamini–Hochberg adjustment for multiple comparisons (q = 0.180). Whole-image, parafoveal, perifoveal, and OD-only FAZ measurements did not differ significantly. Six of seven archived *FBN1* reports documented a variant, while one was negative within its assay scope. These observations are preliminary and hypothesis-generating; they do not demonstrate diagnostic feasibility, clinical utility, disease prediction, or an individual-level genotype–OCTA relationship.

The direction of the foveal estimate is broadly consistent with observations in genetically confirmed Marfan syndrome, although direct comparison is limited by fundamental differences in study design. Our study was organized according to lens-dislocation phenotype and was not designed to test individual-level genotype–OCTA or cardiovascular–OCTA associations. It therefore provides a complementary exploratory observation rather than evidence of a shared causal mechanism. On the other hand, Kinori et al. described distinctive ocular biometric characteristics in Marfan syndrome [22]. Di Marino et al. reported reduced superficial and deep retinal capillary plexus and choriocapillaris vessel density, together with associations between selected ocular and cardiovascular measurements [3]. Rezar-Dreindl et al. also identified differences in superficial-plexus vessel density and FAZ-related parameters [20]. More recently, Kéki-Kovács et al. examined genotype, retinal imaging, and cardiovascular parameters in 39 genetically characterized participants with Marfan syndrome [21]. Genotype-related differences were observed mainly in retinal thickness, whereas lower superficial and deep vessel density was reported in participants with a history of aortic surgery [21].

Unlike these cohorts, our study was organized by lens-dislocation phenotype rather than by a pre-established genetic diagnosis. This phenotype-first design reflects a real-world scenario in which ectopia lentis may prompt systemic and genetic evaluation. OCTA was examined here as an exploratory research measure; the present data do not support its use for clinical triage, diagnosis, prognosis, or individualized risk estimation. The lack of statistical significance after multiplicity correction underscores the need for larger, biometry-adjusted studies to determine whether the directional signal observed here represents a true biological effect or a chance finding amplified by small sample size.

### The confounding role of ocular biometry

Ocular biometry interpretation is substantially constrained by the between-group differences. LD+ participants had more negative spherical equivalent (SE) values and longer axial length (AL) than LD− participants. Both refractive status [17] and AL-dependent ocular magnification [18] can influence OCTA-derived vessel-density and FAZ measurements. Because the cohort was too small for reliable multivariable adjustment or magnification-corrected sensitivity analyses, the contributions of lens-dislocation phenotype and ocular biometry cannot be separated. Any observed OCTA difference may therefore reflect either factor, or both.

The FAZ results require similar caution. The absence of a statistically significant difference should not be interpreted as evidence of equivalence. FAZ measurements were available only for the right eye and only in 14 participants, limiting precision and preventing bilateral participant-level averaging. FAZ varies with demographic and foveal characteristics and is sensitive to AL-related scaling, image quality, and segmentation [19]. Annular vessel-density or other FAZ-adjacent metrics may provide complementary information [18], but the present study was neither designed nor powered to compare the performance of candidate OCTA biomarkers.

### Real‑world *FBN1* testing and variant reporting

The seven archived *FBN1* reports provide a snapshot of real-world clinical genetic testing rather than a systematically characterized molecular cohort. Contemporary *FBN1* studies use heterogeneous testing strategies, including full-gene sequencing, multigene next-generation sequencing panels, and follow-up familial segregation analyses, which makes direct comparison across report types difficult [23,24]. Even in well-characterized Marfan cohorts, some clinically affected individuals remain mutation-negative or require additional methods beyond initial screening, underscoring the limits of assay scope and technical sensitivity [25,26]. Accordingly, the six variant-positive and one report-negative results in this archive should not be interpreted as diagnostic yield, and the small number of reports, related individuals, and assay heterogeneity preclude meaningful genotype–phenotype or genotype–OCTA inference [27].

Variant-level interpretation also requires caution. *FBN1* variation is highly heterogeneous, and many variants are unique to an individual or family, limiting stable genotype–phenotype generalization [28]. Although truncating and cysteine-altering variants are recognized disease mechanisms, interpretation of any specific variant depends on standardized nomenclature, population frequency, segregation, phenotype, and formal classification evidence [29,30]. Accordingly, the source-report notations c.4349G > C (p.Cys1450Ser) and c.5368C > T (p.Arg1790*) are reproduced as issued. This study did not review raw sequence data, standardize transcripts, or independently apply ACMG/AMP or *FBN1*-specific criteria [31–33]. The archived reports therefore support only a descriptive account of prior clinical testing and cannot establish variant pathogenicity, diagnostic yield, genotype–phenotype associations, or the proposed biological pathway.

### A hypothesis‑generating mechanistic framework

The directional foveal finding can be considered only within a hypothesis-generating three-tier evidence framework (Fig 1). In this framework, zonular weakness and possible retinal microvascular variation are viewed as parallel research observations linked by biological plausibility, rather than as evidence that lens dislocation directly causes posterior-segment vascular remodeling or that the present study demonstrated a shared molecular mechanism.

A more precise interpretation of Fig 1 is as a three-tier framework rather than a single causal model. First, the ocular structural tier is well established: fibrillin-1 is a major constituent of the ciliary zonule, and depletion of fibrillin-1 in the non-pigmented ciliary epithelium weakens zonular fibers, reduces tensile strength, and leads to ectopia lentis in mice. Second, the vascular tier is biologically plausible but less direct. Fibrillin-rich microfibrils interact with latent TGF-β-binding proteins and help regulate extracellular sequestration and activation of latent TGF-β complexes [34,35]. Across Marfan models and human aortic studies, disturbed fibrillin-1/TGF-β signaling has been linked to abnormal Smad2, ERK, and p38 signaling, endothelial dysfunction, extracellular-matrix remodeling, smooth-muscle phenotypic change, and aneurysm progression [36,37]. However, this literature is not uniform: several studies indicate that TGF-β effects are stage-dependent and context-dependent, that canonical signaling may be reduced in some vascular cell populations despite TGFB1 upregulation, and that physiologic TGF-β signaling can be protective in early aortopathy or other aneurysm models [36,38]. Third, the retinal tier remains suggestive but unproven. Retinal studies show that fibrillin-1 localizes around mouse retinal arterioles and contributes to arteriolar integrity, and later work demonstrated a role at the angiogenic front in endothelial sprouting and tip-cell specification, making a retinal microvascular pathway biologically plausible alongside prior human OCTA observations. Even so, the bridge from this experimental biology to the OCTA pattern observed here remains untested: most mechanistic support comes from systemic, aortic, cerebrovascular, zebrafish, or iPSC-based models rather than the human retina [34,36,41,42]. Retinal or circulating TGF-β activity, extracellular-matrix remodeling, and endothelial function were not measured in this study, so Fig 1 should be read strictly as a hypothesis-generating synthesis that distinguishes established zonular biology, supportive experimental vascular evidence, and the still-unproven link to human retinal OCTA measurements.

### Implications for future phenotype-stratified research

The present findings do not establish OCTA as a diagnostic or prognostic test and do not show that OCTA combined with *FBN1* testing predicts systemic disease progression. Instead, they define a research framework for prospective phenotype-stratified studies using protocol-locked serial imaging, standardized molecular testing, ocular-biometry adjustment, and prospectively collected systemic outcomes. Clinical use or individualized risk estimation would require independent validation in substantially larger cohorts.

### Limitations

The principal limitations are the small, retrospective, availability-based sample and the absence of a complete screening denominator or exclusion log; selection bias therefore cannot be quantified, and consecutive inclusion cannot be assumed. OCTA interpretation is constrained by marked between-group differences in SE and AL, the absence of adjusted or magnification-corrected analyses, incomplete eye-level data, OD-only FAZ availability, restriction to the SCP, and single-grader image-quality assessment. The genetic review was confined to archived reports from the LD+ subgroup and was further limited by related participants, heterogeneous assay scope, and report-transcribed nomenclature without raw-data reanalysis or contemporary reclassification. Accordingly, these findings are hypothesis-generating and cannot establish causality, diagnostic yield, genotype–phenotype or genotype–OCTA associations, or individual risk.

## Conclusion

This exploratory study provides a preliminary participant-level OCTA comparison and a descriptive account of archived *FBN1*-report heterogeneity. Its main contribution is to define a reproducible phenotype-stratified research question rather than to establish a diagnostic workflow. Future studies should recruit larger cohorts, account for ocular biometry and image magnification, and apply standardized genomic testing—including comprehensive *FBN1* analysis with deletion/duplication detection or, where appropriate, exome or genome sequencing—to enable adequately powered genotype–phenotype and genotype–OCTA analyses.

## Supporting information

S1 File Data dictionary for publicly shareable variables and derived measures. (XLSX)

S2 File Aggregate results underlying Tables 1–3. (XLSX)

S3 File Analysis code used to reproduce the aggregate results. (PY)

S4 File README describing the supporting files and controlled-access request procedures. (TXT)
